# Rationale and design of a prospective study on the first integrated PET/dual-energy CT system for staging and image-based radiation therapy planning of lung cancer

**DOI:** 10.1186/s41747-018-0047-4

**Published:** 2018-07-04

**Authors:** Carlo N. De Cecco, Philip Burchett, Marly van Assen, James Ravenel, Samuel Lewis Cooper, Hong Li, Marques L. Bradshaw, William J. Rieter, U. Joseph Schoepf, Leonie Gordon

**Affiliations:** 1 Department of Radiology and Radiological Science, Medical University of South Carolina, 25 Courtenay Drive, Charleston, SC USA; 2 University of Groningen, University Medical Center Groningen, Center for Medical Imaging - North East Netherlands, Groningen, The Netherlands; 3 Department of Radiation Oncology, Medical University of South Carolina, Charleston, SC USA; 4 Department of Public Health Sciences, Medical University of South Carolina, Charleston, SC USA; 5 Department of Radiology, Vanderbilt University Medical Center, Nashville, TN USA

**Keywords:** Positron emission tomography (PET), Dual-energy computed tomography (DECT), Lung cancer, Tumor staging, Image-guided radiation therapy (IGRT)

## Abstract

**Background:**

The aim of this prospective study is to investigate the diagnostic performance of integrated positron emission tomography (PET) /dual-energy computed tomography (DECT) imaging in determining the thoracic nodal status of patients with small-cell lung cancer (SCLC) or non-small-cell lung cancer (NSCLC) and the resulting impact on target volume delineation for radiation therapy planning.

**Methods:**

This is a single-center prospective study including approximately 50 patients with suspected or confirmed SCLC or NSCLC, referred for a PET study. All patients will be examined on a clinical PET/DECT system, where a dual-energy detector was recently installed. The patient will be placed in the system 70 min after the administration of 5 MBq/kg of ^18^F-fluorodeoxyglucose (^18^F-FDG). Then, DECT will be acquired after the injection of 100 mL of iodine contrast medium. A PET scan will be acquired from the top of the skull through the inguinal region. Data analysis will be performed on the PET, CT, and iodine map datasets. Information regarding tumor detection, adenopathies, and radiation therapy planning will be assessed based on all three sets of images by two experienced radiologists.

**Conclusion:**

The results will add insights into the advantages of using PET/DECT for lung cancer staging and for image-guided radiation therapy.

**Trial registration:**

ClinicalTrials.gov, NCT03146117. Registered on 9 May 2017.

## Key points

- PET/CT is the established standard for planning lung cancer radiation therapy
- PET/DECT may improve accuracy for lung tumor detection and nodal staging
- PET/DECT may improve accuracy for radiation therapy planning

## Background

^18^F-fluorodeoxyglucose (^18^F-FDG) positron emission tomography (PET) plays an important role in the staging of small-cell lung cancer (SCLC) and non-small-cell lung cancer (NSCLC). Multiple studies have demonstrated the utility of PET for improving staging accuracy, with respect to the mediastinum and distant metastatic involvement compared to computed tomography (CT) alone [1–5]. Additional improvements in staging accuracy are encountered when functional PET imaging is combined with CT in integrated and dedicated PET/CT systems [6, 7]. Furthermore, PET/CT should be used for image-guided radiation therapy (IGRT) planning because it images tumor extent with greater accuracy than CT alone increasing normal tissue sparing from futile irradiation, especially in case of atelectasis, and provides a better assessment of involved nodes [8, 9].

Despite the higher accuracy of PET/CT, the limitations of PET should be recounted. The rate of false-negative lymph node station assessment in patients with NSCLC candidates to radiation therapy is reported to be in the range of 5–10% [10]. Small lesions (< 1 cm) are difficult to identify and in the setting of elevated blood glucose the consequent decrease in tumor ^18^F-FDG-PET uptake may cause a false-negative result [11]. False-negative scans can also occur soon after chemotherapy [9]. Regarding IGRT planning, the margins of PET-detected lesions can appear blurred due to respiration. Moreover, a large degree of uncertainty exists regarding the most appropriate standard uptake value (SUV) threshold cut-off value that would ideally define a PET target volume in NSCLC treatment planning [12].

Dual-energy CT (DECT) is a relatively new imaging technique, whose basic principle is the application of two distinct energy settings with the transition from CT density-based imaging to material-specific or spectral imaging [13]. The possibility to generate iodine map images, which enable an accurate quantification of iodine uptake within a lesion could represent a reliable quantitative biomarker for tumor detection and lymph node characterization [14].

In the literature, the advantages of combined PET/DECT imaging in determining the nodal status of patients with SCLC or NSCLC as well as in guiding radiation therapy planning have not been investigated. Thus, this study has been designed to assess the role of the first integrated PET/DECT system for staging and IGRT planning in lung cancer [15, 16].

## Methods

### Study design and patient population

**Fig. 1:**
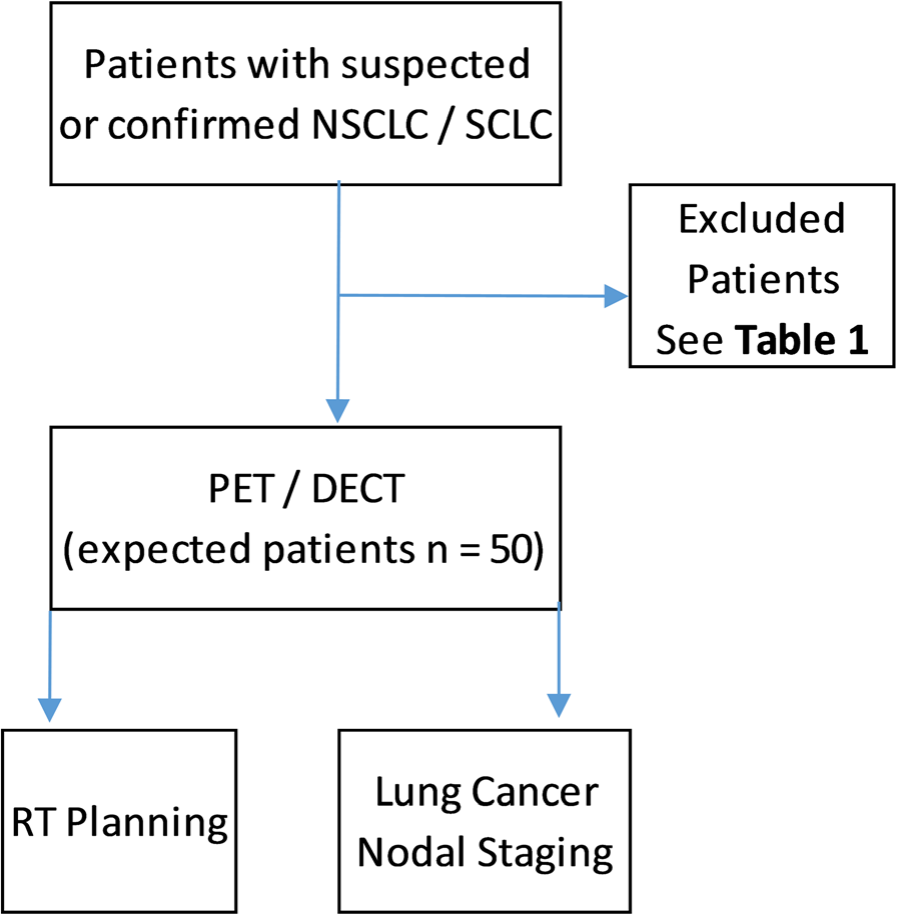
Study design *flowchart*. *SCLC* Samall-cell lung cancer, *NSCLC* Non-small-cell lung cancer, *PET/DECT* Positron emission tomography/dual-energy computed tomography, *RT* Radiation therapy

This is a prospective, single-center study designed to evaluate a novel PET/DECT system in patients with suspected or confirmed SCLC or NSCLC who are referred to undergo a clinically indicated PET/CT for staging. We plan to recruit approximately 50 individuals either male or female, in the age range of 18–90 years, with suspected or confirmed SCLC or NSCLC referred to undergo a clinically indicated ^18^F-FDG-PET/CT examination (Fig. 1).

Clinical staging will be performed using the 8^th^ edition TNM classification system by the American Joint Committee on Cancer [17] based on CT, PET/CT, and brain magnetic resonance, as clinically indicated. Pathologic staging may incorporate percutaneous biopsy, endobronchial ultrasound, and surgery determined by accepted clinical guidelines. Institutional Review Board review and approval will be obtained before commencing enrollment. The inclusion and exclusion criteria are summarized in Table 1.

**Table 1:** Patient eligibility criteria

	Inclusion criteria		Exclusion criteria
1	Subject must have a suspected SCLC or NSCLC	1	Subject is a pregnant or nursing female
2	Subject must be aged 18–90 years	2	Subject is in acute unstable condition
3	Subject must have been referred for a clinically indicated PET/CT	3	Subject is unwilling to comply with the requirements of the protocol
4	Subject must provide written informed consent before any study-related procedures being performed	4	By testing (serum or urine βHCG) within 24 h before contrast, agent administration, or by surgical sterilization, or post-menopausal, with minimum one-year history without menses
5	Subject must be willing to comply with all clinical study procedures	5	Subject has an allergy against iodinated contrast agents and cannot be premedicated
		6	Subject has impaired renal function (eGFR < 30 mL/min × 1.73 m^2^)
		7	Subject has an acute psychiatric disorder or is cognitively impaired
		8	Subject is using or is dependent on substances of abuse

*SCLC* small-cell lung cancer, *NSCLC* non-small-cell lung cancer, *PET/CT* positron emission tomography / computed tomography, *βHCG* β-human chorionic gonadotropin, *eGFR* estimated glomerular filtration rate

### Study objectives

The overall goal of this project is to investigate the diagnostic performance of integrated PET/DECT imaging in determining the thoracic nodal status of patients with SCLC or NSCLC and its impact on target volume delineation for IGRT planning.

### Imaging protocol

**Fig. 2:**
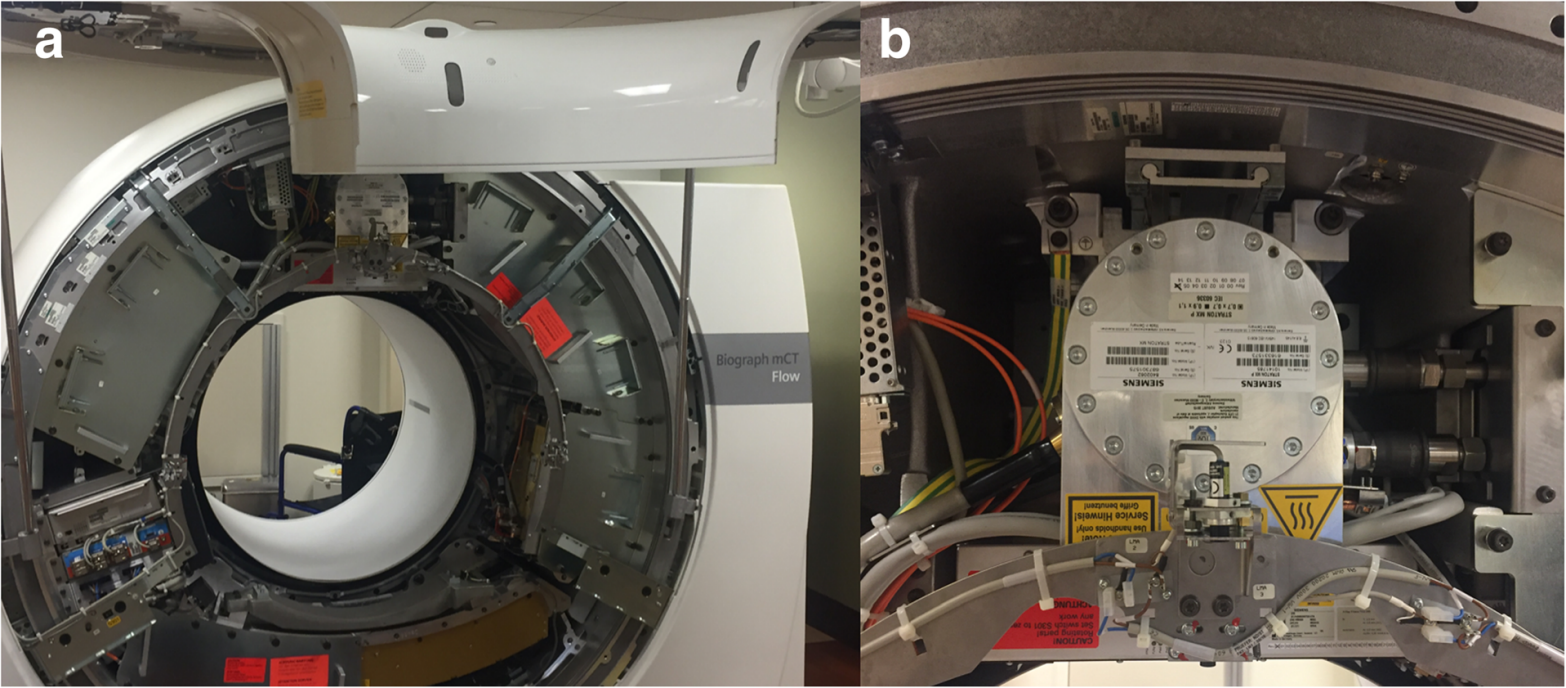
**a** Integrated PET/DECT system. **b** Particular of the TwinBeam dual-energy system

All patients will be examined on a clinical PET/CT system (Biograph mCT, Siemens Healthineers, Forchheim, Germany), where a dual-energy detector (TwinBeam Dual Energy, Siemens Healthineers, Forccheim, Germany) has been installed (Fig. 2). Patients will be placed in the supine position with both arms raised above their head. Our standard clinical PET/DECT protocol includes a non-breath-hold contrast-enhanced DECT scan with the arms down for attenuation correction, acquired from the top of the skull through the inguinal region, followed by a shallow breath DECT scan of the chest, acquired from the pulmonary apex through the diaphragm, with the arms up. For contrast injection, we will use an automatic double-head power injector to administer a total of 100 mL of non-ionic iodinated contrast material at a flow rate of 3.0 mL/s, followed by a 20-mL saline flush.

The patient will be placed in the system 70 min after the administration of 5 MBq/kg of ^18^F-FDG. The patient fasting glucose level will be measured before the injection. A PET scan will be acquired from the top of the skull through the inguinal region. A three-dimensional (3D) emission scan of the same areas will be acquired.

**Fig. 3:**
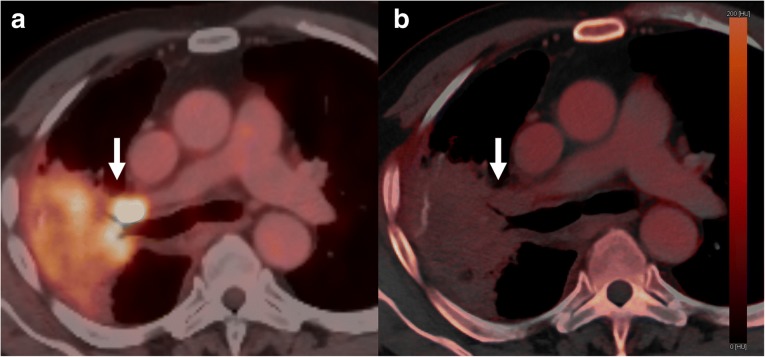
PET and DECT images comparison in a patient with lung cancer and metastatic right hilar adenopathy. **a** Fused PET/CT dataset showing metabolic uptake of the right hilar adenopathy and lung mass (*arrow*). **b** Dual-energy iodine map dataset showing iodine uptake in the same region (*arrow*)

Axial CT datasets will be reconstructed using a 3-mm section thickness and increment. Low-energy 120 kVpSn (tin filtration) and high-energy 120 kVpAu (gold filtration) datasets [18] will be loaded into a dedicated dual-energy post-processing workstation (Siemens Multi-Modality Workplace MMWP, Siemens Healthineers, Forccheim, Germany) and a mixed standard 120-kV dataset and iodine-map dataset will be generated by using an application available on the workstation (Dual Energy Syngo, Siemens Healthineers, Forccheim, Germany). Twin-beam datasets were used to generate linearly blended and noise-optimized virtual monoenergetic images at 50 keV using iterative reconstruction strength 3 (Safire, Siemens Healthineers, Forccheim, Germany). To generate iodine map images, standard soft-tissue and fat attenuation values in default settings will be used (soft tissue and fat attenuation of 60/55 HU and − 110/− 87 HU at low/high kVp, respectively); beam-hardening correction, organ contour enhancement, and resolution enhancement will be also applied (Fig. 3).

### Data analysis

#### Tumor detection

Before analysis, images from each patient will be separated into three groups (PET, CT, iodine-map) corresponding to three different datasets. Images will be all reviewed on a picture archiving and communication system workstation. Two radiologists with > 5 years of experience will independently analyze the number of pulmonary lesion(s), mediastinal and hilar adenopathies, and distant metastases in the three datasets in separate reading sessions. Lymph nodes will be considered to be involved if they demonstrate a SUV > 3.0 on PET, a short axis ≥ 10 mm in diameter on CT, or a iodine reduction on the iodine-map (mean iodine concentration < 2.5 mgI/mL). For each lesion, the CT diameter, CT attenuation, iodine concentration, and SUV will be measured using a region of interest drawn by the radiologist. Radiologists will be blinded to patient identity, lesion number, and the results of other imaging studies. To avoid recall bias, the time interval between each of the reading sessions will be at least two weeks. After this blind evaluation, the study coordinator will review all images and match the imaging findings with the lesions marked by each reader in each dataset. The reference standard will be the complete evaluation of the PET/CT performed separately from the individual components above. Discrepancies between radiologists will be handled in consensus.

#### CT simulation for radiation therapy planning

Patients who will receive radiation therapy for locally advanced lung cancer will be simulated with immobilization to assure reproducibility of the setup. Each patient will be positioned in an immobilization device in the treatment position on a flat Table. A volumetric planning CT will be performed with no more than 3-mm thickness from the level of the cricoid cartilage through the entire liver volume. Four-dimensional radiation planning is required and can include design of the planning target volume (PTV) to account for primary and nodal tumor motion during free breathing or the more limited excursion during deep inspiration breath-hold with a real-time position management system (RPM, Varian Medical System, Palo Alto, CA, USA).

#### Virtual simulation and 3D treatment planning

The fused images will be transferred to a dedicated workstation. The gross tumor volume (GTV) will be defined by the planning physician as all known gross disease (gross tumor volume, pulmonary [GTV-P]) and involved mediastinal and hilar nodes (GTV-N) as demonstrated on the treatment planning scans. Two GTVs contours will be outlined by the same radiotherapist for each patient. The first volume will be defined from the anatomic data provided by PET/CT and the second volume will be defined from the anatomic data provided by PET/DECT. The GTV-N will include only those lymph nodes considered to be involved. As already stated above, lymph nodes will be considered to be involved in PET/CT and in PET/DECT when they demonstrate an increased ^18^F-FDG uptake (SUV > 3.0) and/or an iodine reduction on the iodine-map (< 2.5 mgI/mL). In addition, any nodes pathologically confirmed to be involved will be included. Non-involved elective nodal regions will be not intentionally targeted.

#### Definition of internal target volume and planning target volume

The internal target volume (ITV) will be used to account for respiratory motion. Four-dimensional CT may be employed, ideally with the acquisition additionally 3D CT images at natural end-inhale and end-exhale. The ITV is the union of the GTV-P and GTV-N contoured on each component 3D CT dataset from the four-dimensional CT acquisition to form the envelope that encompasses the motion of the GTV for a complete respiratory cycle [19–21]. The internal margin will be 0.5 cm in the superior-inferior direction and 0.3 cm in the axial plane. The setup margin will be 0.5 cm in all directions. No margin reduction will be allowed even when using IGRT. The final PTV will be obtained by expanding the ITV by the setup margin.

#### Radiation therapy dose prescription

The total dose will be 60 Gy in 30 fractions of 2 Gy. The treatment plan will be normalized so that 95% of the PTV is covered by the prescription dose. The maximum dose to 0.1 cm^3^ should not exceed 110%. 3D conformal radiation therapy or intensity modulated radiation therapy will be used. All radiation doses will be calculated with heterogeneity corrections taking into account the density differences within the irradiated volume (e.g. air, soft tissue, or bone).

### Statistical analysis

All analysis will be performed using Statistical Analysis System (SAS 9.4, Cary, NC, USA). This is a pilot study with a convenience sample of 50 participants for Aim 1 (approximately 5–10 lymph nodes per patient) and the first 20 participants who undergo radiation therapy treatment for Aim 2. The analysis will include node-based analysis and patient-based analysis.

#### Aim 1: node-based analysis

Descriptive statistics will summarize the location and the number of positive cases for each lymph node using CT (where the test positive is defined as a lymph node short axis diameter > 1 cm), PET (where the test positive is defined as that SUV > 3.0), and DECT (where the test positive is defined as that the iodine is < 2.5 mgI/mL). To address clustering, we will perform a multilevel model test. The diagnostic power will be summarized by the area under the curve at receiver operating characteristic analysis (AUC), comparing CT and PET, CT and DECT, as well as PET and DECT. Multilevel models will be used to estimate the probability of a positive, which will be used to estimate the empirical AUC. The following additional analysis will be done to further understand the performance of CT, PET, and DECT. First, the Cohen’s κ statistics will be used to assess the concordance between CT and PET, DECT and PET, and DECT and CT. Second, for patients with known disease status, the AUC will be evaluated comparing CT and PET, and DECT and PET, using the complete PET/CT examination and the pathological specimens, if available, as a reference standard for lesion detection and characterization.

#### Aim 2: patient-based analysis

We will evaluate the impact of DECT and FDG-PET image fusion on treatment planning in 20 patients out of the 50 recruited who will receive radiation therapy at our institution. Descriptive statistics will be used to summarize GTV measure. A Wilcoxon Signed-Rank test will be used to explore the statistical difference of the above measurements between PET/CT and PET/DECT.

## Discussion

This prospective clinical trial has been designed to assess the role of the first integrated PET/DECT system for staging and IGRT planning in lung cancer. In detail, we plan to investigate the role of PET/DECT in determining the nodal status of SCLC and NSCLC, and IGRT planning [22, 23].

We speculate that the accurate quantification of iodine uptake obtained with the DECT could improve the morphological delineation of lesions, differentiating normal tissue and atelectasis from lung cancer [22, 24], allowing a smaller volume of lung to be treated with IGRT. In addition, recent data suggest that it could improve the detection of pathological lymph nodes [21], which is particularly relevant when SUV results are borderline [25, 26].

Knowledge gained during this study may potentially allow for an improvement in PET/CT accuracy for lung tumor and thoracic adenopathy detection, and for a more accurate definition of the treatment target decreasing the radiation dose delivered to normal tissue.

## Abbreviations

^18^F-FDG: ^18^F-fluorodeoxyglucose
3D: Three-dimensional
CT: Computed tomography
DECT: Dual-energy computed tomography
GTV: Gross tumor volume
GTV-N: Gross tumor volume, nodes
GTV-P: Gross tumor volume, pulmonary
IGRT: Image-guided radiation therapy
ITV: Internal target volume
NSCLC: Non-small-cell lung cancer
PET: Positron emission tomography
PVT: Planning target volume
SCLC: Small-cell lung cancer
SUV: Standard uptake value
